# Complementarity of formal and informal actors and their networks in support of vulnerable populations in informal settlements: Governance diaries approach

**DOI:** 10.3389/fpubh.2022.1043602

**Published:** 2023-01-27

**Authors:** Ivy Chumo, Caroline Kabaria, Alex Shankland, Emmy Igonya, Blessing Mberu

**Affiliations:** ^1^African Population and Health Research Center (APHRC), Nairobi, Kenya; ^2^Institute of Development Studies (IDS), Brighton, United Kingdom

**Keywords:** formal and informal arrangements, complementarity, informal settlements, marginalized and vulnerable groups, qualitative research, Kenya

## Abstract

**Introduction:**

Beyond several interests and speculations on the relationship between formal and informal actors and their networks in support of vulnerable populations, most studies do not conclusively establish whether the two types of support are substitutes or complements. While informal care and formal care may be substitutes in general, they are complements among the vulnerable groups. Despite how some studies have described complementarity, further insights on the synergy between formal and informal actors and networks are needed to pinpoint how to maximize policy and interventions to alleviate the challenges facing vulnerable groups in informal settlements.

**Methods:**

We conducted an ethnography using governance diaries with 24 participants in Korogocho and Viwandani informal settlements in Nairobi, Kenya. The governance diaries approach involved conducting bi-weekly governance in-depth interviews (IDIs) with study participants for 4 months, complemented with observations, reflections, participant diaries and informal discussions. We used framework analysis approach.

**Findings:**

Informal actors identified include family, neighbors, friends, community groups and community members, and their direct networks. Formal actors on the other hand included government institutions, individuals and authorities that make policies and rules and their desired and possible networks. Both the formal and informal actors and their networks had complementary roles that were beneficial to the vulnerable populations living and working in informal settlements. The complementarities between formal and informal actors and networks in supporting vulnerable groups were portrayed in roles and responsibilities to the vulnerable groups; rules, regulations and governance in supporting vulnerable groups; knowledge, skills and dynamic workforces among formal and informal actors and their networks; information flow on health and wellbeing to the vulnerable populations; transition of actors in supporting vulnerable groups; availability, access and involvement of formal and informal actors and networks to support vulnerable groups. The complementarities allowed for maximum support of the vulnerable populations than otherwise.

**Conclusion:**

We conclude that informal social support is needed regardless of the availability of formal social support. Moreover, a combination of formal and informal actors and related networks are essential to support vulnerable persons. Formal actors should establish, support, or maintain the informal actors and related networks through goodwill and sundry incentives as a vital dimension of building with local community structures and enhancing inclusion, participation and ownership of policy and program interventions by marginalized and vulnerable groups.

## Introduction

Although several authors have explored the relationship between formal and informal actors and their networks in support of vulnerable populations (1), most studies do not definitively establish whether the two types of support are substitutes or complements (2, 3). While informal care and formal care may be substitutes in general, they are complements among vulnerable groups (4). Despite how some studies have described complementarity, further insights on the synergy between formal and informal actors and networks are needed (2, 5, 6). Formal actors are institutions, individuals and authorities that make policies and rules and their actions are embedded in formal institutions (7). Informal actors are individuals or groups who have no constitutional mandate and do not have formal rules but still have an influence on service provision (1). In the near absence of formal actors and networks, informal settlements are characterized as ungovernable spaces lacking formal services (7, 8), but the urban poor in such settlements are resilient and often create their own systems to fill gaps in service delivery (9, 10). The service gaps are mostly filled by informal actors (10, 11), often seen with adversarial lens from the point of view of law and order (1, 12). Yet they constitute part of the social fabric and provide complimentary or lacking services and influence, sometimes better than formal actors and networks within informal settlements (13).

Complementary model of support takes into account support from both formal and informal actors and networks through compensation and/or supplementary functions by the actors (5, 6). According to this model, formal actors and networks are accessed when crucial elements of the informal actors and networks are lacking or when there is great need (i.e., greater illness or disability) and the informal actors are unable to provide the required support (3, 5). Most support for vulnerable groups is provided informally by family and friends at home (14). Yet, increasing changing family structures (i.e., single-parent households, decreasing family size and increasing women participation in labor force) means that a growing number of vulnerable groups require and will use formal support services (15, 16). The two types of support can occur simultaneously or can precede or follow each other (3, 17). Even when the two types of care occur simultaneously, informal care may not be a substitute for formal care but a complement for higher skilled tasks (18). A single actor and network may not be able or willing to provide care, suggesting that informal and formal care are not substitutes but compliments in many cases (19), thus the need for more insights on endogeneity inherent in formal and informal actors and networks so as to uncover the complementarity of formal and informal actors and networks (19, 20).

In spaces where only informal support is provided or where only formal support is provided, the vulnerable populations tend to have deteriorated support on health and wellbeing services (21, 22). Where formal and informal service providers have specialization or overlap, evidence point to the vulnerable being better served (5, 6). When there is an overlap, social support received by vulnerable groups seem to be key (23, 24), irrespective of whether it comes from formal or informal sources (2). However, the synergy of formal and informal actors and their networks in support of vulnerable populations in informal settlements is often missed out in the policy discourse and inputs to interventions. Consequently, further insights on the synergy between formal and informal actors and actions are needed to pinpoint how to maximize policy and practice inputs to alleviate the challenges of vulnerable groups in informal settlements (6). Indeed, the linkages between informal-formal actors and networks in informal settlements should be further uncovered for joint utilization of informal and formal actors and networks (25, 26). In Nairobi's informal settlements, informal actors and their networks have engaged and collaborated with the state complementarily to support the vulnerable populations living and working in informal settlements (25, 27). Strategic complementarity enhances a multiplier effect, benefits, expertise and knowledge in supporting vulnerable populations (27). Yet, it is unclear how formal and informal actors and their networks work together to impact the health and wellbeing of vulnerable populations in the informal settlements (28). In seeking to address these knowledge gaps, this study contributes to understanding the following two questions: first, what are the benefits of formal and informal actors? Second, how do formal and informal actors complement each other for the benefit of vulnerable groups?

## Literature review

This section presents literature on informal settlements in Kenya; marginalized and vulnerable populations, formal and informal actors and networks, including complementarity of the actors.

### Informal settlements in Kenya

Informal settlements are unplanned sites that are not compliant with authorized regulations (29). The widespread growth of informal settlements in urban centers in Kenya has become a central debate in urbanization during the last two decades (30). Yet, the lag by the Kenyan government to improve informal settlements and at least to provide the minimum support on basic requirements and services has led to unimaginable suffering among residents (31). This is coupled by the fact that the government has had the history of failing to recognize the growth and proliferation of informal settlements and thus excludes the urban poor from the rest of the city's development plan (31, 32). While constitutional and attitudinal changes are observable, it is hoped that advocating for the urban poor, particularly marginalized and vulnerable groups would help change the course of events in informal settlements in Nairobi, Kenya.

### Vulnerable and marginalized population

Vulnerability refers to the conditions determined by physical, social, economic and environmental factors or processes, which increase the susceptibility of an individual or community to the impact of hazards (33, 34). A vulnerable group is therefore a population that has some specific characteristics that make it at higher risk of falling into poverty (33), those who by virtue of gender, ethnicity, age, physical or mental disability, economic disadvantage, or social status may be more adversely affected in the community than others and may be limited in their ability to claim or take advantage of assistance and related development benefits (33, 35). Marginalization generally describes the overt actions or tendencies of human societies whereby those perceived as being without desirability or function are removed or excluded (i.e., are “marginalized”) from the prevalent systems of protection and integration, so limiting their opportunities and means for survival (33). A vulnerable and marginalized individual/group is defined as a group that in a particular context because of its relatively small population or for any other reason, has been unable to fully access basic amenities and participate in the integrated social and economic life as a whole (33, 36).

Children including child-heads of households (CHHs) are more vulnerable to health and wellbeing challenges (36, 37). Three elements of challenges for a CHHs are biological and physical challenges; strategic challenges (i.e., children's limited levels of autonomy and dependence on adults); and institutional invisibility due to a lack of voice in policy agendas (38). Older people face challenges including a lack of access to regular income, work and health care; declining physical and mental capacities; and dependency within the household (35). Without income or work, older people tend to depend on others for their survival and are usually vulnerable in informal settlements where their caregivers also have health and wellbeing challenges (11, 39). Persons with disability (PWD) are often (sometimes incorrectly) assumed to be unable to work and hence increasing their vulnerabilities to health and wellbeing challenges (40). Evidence shows that PWD have higher rates of poverty, and face physical, communication and attitudinal barriers, and a lack of sensitivity or awareness about their circumstances and situations (41). With the clear understanding of different vulnerable and marginalized population, It is widely acknowledged that the populations have unique basic needs from the general population and are harder hit, both in the short and long term, when there is no action taken and thus there is a need for formal and informal actors to take action for their livability (10, 38).

### Formal and informal actors and networks

Formal actors are groups or individuals with defined and structured guidelines, whereas informal actors have undefined guidelines (42, 43). Informal actors play a key role to persist and retain legitimacy in informal settlements (2). Informal actors are diverse and may include community members or customary local governance institutions. Often, they fulfill some of the functions expected of the state, yet they are understudied, particularly with regard to how they complement formal actors (6). Within the context of social relationships, complementarity mostly refers to the premise that people tend to seek out other individuals with characteristics that are different yet complementary from their own (a concept sometimes called negative assortative mating by organizational theorist) (6). Philosophers have argued that formal and informal support systems cannot exist in strong form in the same society (17, 44). In this article, the authors argue and demonstrate that such perspectives fail to consider the complementarity of the two systems and the necessity of both for the completion of most tasks, including service provision for the marginalized and vulnerable populations.

## Methods

The study is reported per a set of standardized criteria for reporting qualitative research (COREQ) (45).

### Study design

This was a qualitative study using the governance diaries method. Governance diaries is an ethnographic approach using more than one method of data collection and where participants make regular records of their daily activities and experiences in relation to actors and who has power in their lives in the provision of social and economic services (46, 47). For this study, governance diaries included in-depth interviews (IDIs), which were informed by participant diaries, informal discussions, participant observations and reflections. Governance diaries are typically used in contexts where there is a need to explore the depth of everyday life, as time allows researchers to spend longer periods in the field for exploration (46, 48).

### Study setting

**Figure 1:**
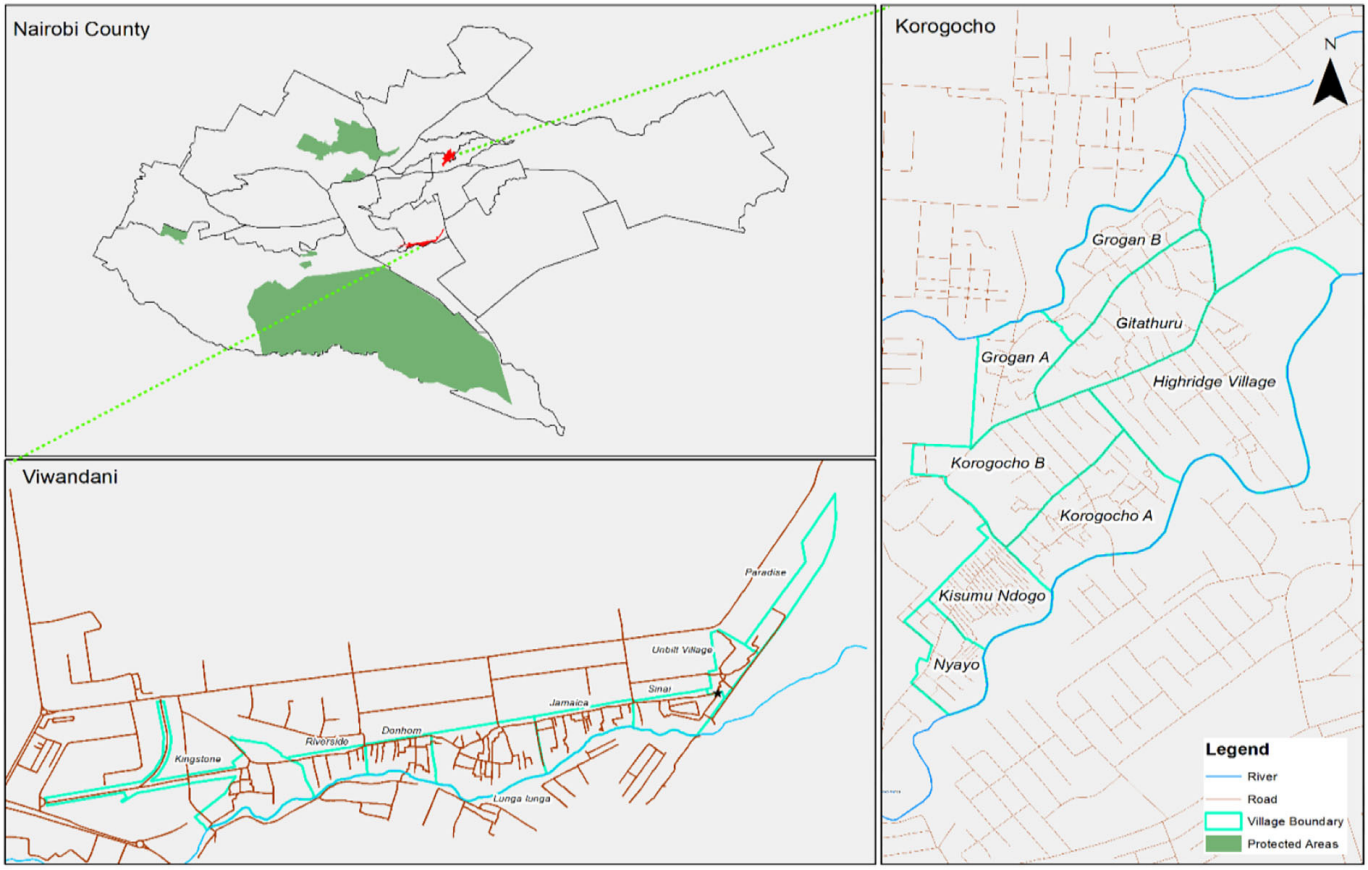
Study sites.

The study was conducted in Korogocho and Viwandani informal settlements in Nairobi, in the areas covered by Nairobi Urban Health and Demographic Surveillance System (NUHDSS) initiated in 2002 by the African Population and Health Research Center (APHRC) (49). Korogocho has a stable and settled population and residents have lived in the area for many years (50), while Viwandani is located next to an industrial area with many highly mobile residents who work or seek jobs in the industrial area (50). A social mapping activity in both settlements identified the most vulnerable groups as persons with disability, older persons and child heads of households. Each of the informal settlements has eight villages, which acted as a guide during the selection of study participants (Figure 1).

### Target population

The population of interest were people with disability, child headed households and older persons.

### Sampling and sample size

We sampled 24 participants comprising four PWD, four CHHs, and four older persons in each of the study sites. We purposively selected the participants who were residents and benefited from health and wellbeing services from at least two villages.

### Data collection process

Community Advisory Committees (CACs) (individuals selected by the community to represent and act as a liaison between the researchers and the community), co-researchers (community members recruited as research assistants because they have a better understanding of the context and with closer rapport with respondents), and the researchers worked together in the recruitment of study participants. We used governance diaries to collect data from January to April 2021 on questions related to the complementarity of formal and informal support actors and related networks for vulnerable populations. Diaries approach entailed IDIs, participant observation, participant diaries, reflection and informal discussions. IDIs were the dominant method and was informed by the other methods. Below is the description of data collection process:

*Informal discussions:* An informal conversation was carried out between the participants and the researchers to find out key insights and to create rapport with the study participants before the IDIs. The discussions were incorporated in the IDIs.

*Reflexive discussions:* Reflective discussions were held between pairs of co-researchers on a daily basis, among the whole group of co-researchers on a weekly basis, and between researchers and co-researchers every 2 weeks, to understand the outcome and determine emerging themes and gaps to be probed during subsequent IDIs and routine observations.

*Observations:* These included observation by the co-researchers, which allowed for a holistic awareness of events as they unfold and as such, enabling more comprehensive understanding of what matters to respondents. We also observed the environment related to our study subjects including observing health and wellbeing services. These observations resulted in photos and insights on what to probe further in the IDIs. The observations were conducted before, during, and after the IDIs to complement the discussions recorded. Reflexive discussions informed the content and concepts for observations.

*Participant diaries*: We provided the study participants with guidelines pasted on the front of a diary. Each participant would write about daily activities related to formal and informal support actors and related networks at their homes, without writing their names. Co-researchers would call participants and conduct impromptu visits to remind participants about diary writing activities.

**Figure 2:**
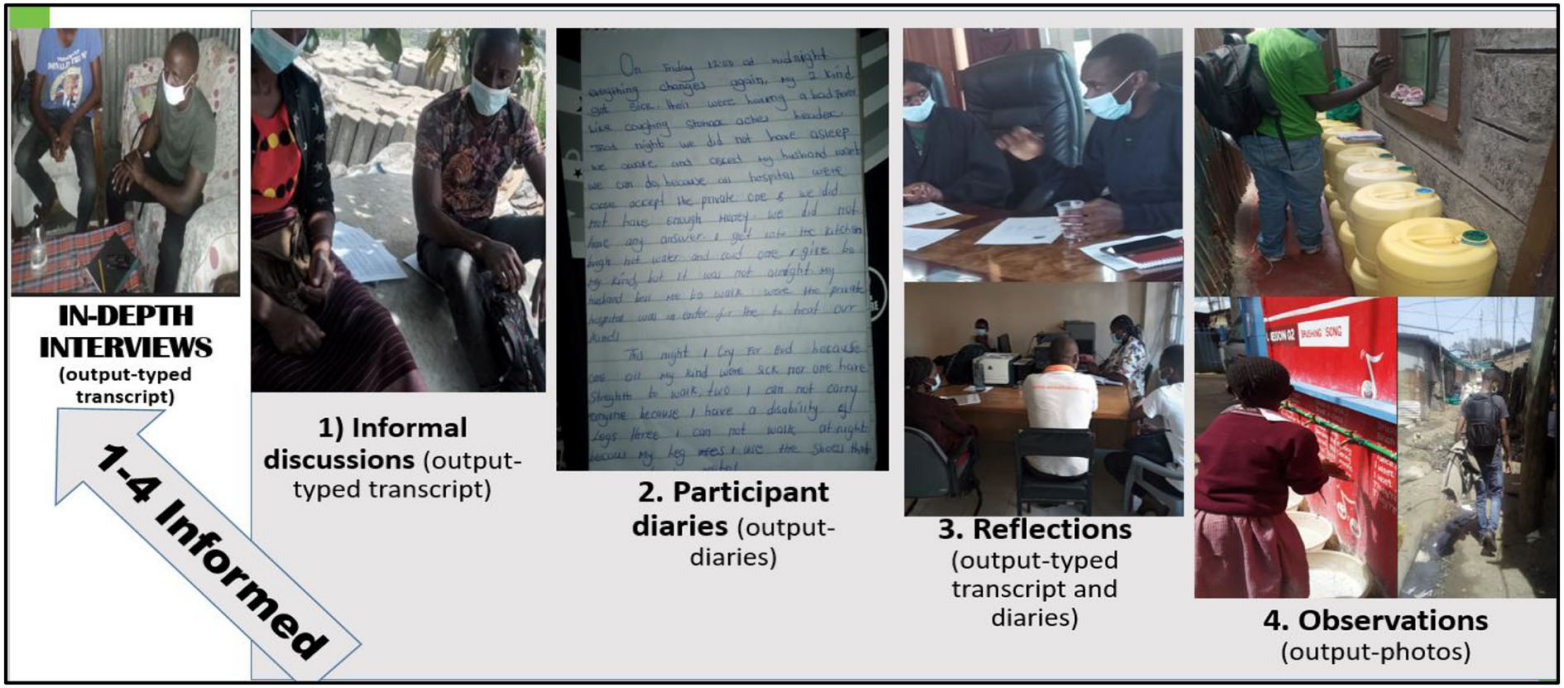
Data collection process.

*IDIs*: We used guides with questions on formal and informal support actors, benefits and complementarity of services and service delivery, concerning the vulnerable populations. IDIs for subsequent visits on the same questions were adapted based on observations, reflections, informal discussion and participant diaries (Figure 2). In-depth discussions between the co-researchers and the study participants were administered in pairs of two co-researchers; one who was moderating the interviews and the second, acting as an observer, note taker and facilitator of the recording of the conversations. We reached saturation in the IDIs during the sixth visit when we approached the fourth month.

The outputs from informal discussions, observations, participant diaries and reflexive discussions informed and enhanced robust probing during IDIs. For example, if the co-researchers observed some ambulance or water pipe burst, during IDIs, they would probe more on the pipe bursts and the ambulance. The multi-pronged ethnographic data collection processes are summarized in Figure 2.

Co-researchers received training for 6 days on the aims of the study, data collection process, data collection tools, and research ethics. We piloted the study tools with one older person, PWD and child head of a household in each of the study sites, followed by a debriefing to assess if the study approach and study tools were well understood by both co-researchers and study participants. The pilot exercise enabled us to adjust the translated guides to concepts understood by the study participants and to estimate the time an interview could take. We excluded participants in the pilot from the main study.

### Data management

Recorded audios from IDIs, reflections and informal discussions were translated and transcribed from Swahili to English and saved as individual Microsoft Word documents. Outputs (Figure 2) were assigned number codes to prepare for analysis and to ensure confidentiality. Outputs that could not be typed (photos from observations and participant diaries) were scanned and saved in a safe folder, as they were used as reference materials during data analysis.

### Data analysis

Transcripts were imported into NVivo 12 software (QSR International, Australia) for coding and analysis. NVivo is a qualitative data management software that can be shared and worked on in groups and facilitate thinking, linking, writing, modeling and graphing in ways that go beyond a simple dependence on coding (51). We used a framework analysis (52). Framework analysis is adopted for research that has specific questions, a pre-designed sample and priory issues (52)^.^ The first step of framework analysis was listening to the recordings to familiarize the researchers with the information related to complementarity of formal and informal actors and networks. To ensure reliability, two researchers (an experienced qualitative researcher and an anthropologist) and five co-researchers, who collected the data participated in the development of a coding framework by reading the outputs imported in NVivo 12 software, participant diaries and photos independently to establish an inter-coder agreement. Once the initial coding framework was completed, the team met to discuss the themes generated and to reach an agreement on themes (Table 1). The two researchers proceeded with coding, charting, mapping and interpretation of transcripts.

**Table 1:** Themes for analysis.

**Major themes**	**Emerging themes**
Formal and informal actors and networks	Formal actors, their networks and benefits Informal actors their networks and benefits
Complementarity of formal and informal actors and their networks in service delivery to vulnerable groups	Complementarity on roles and responsibilities Complementarity on rules, regulations and governance Complementarity on knowledge, skills and family dynamics Complementarity on information flow to the vulnerable Complementarity on transition of actors Complementarity on availability and access of actors Complementarity on involvement

### Ethical considerations

The study was approved by the AMREF Health Africa's Ethics and Scientific Review Committee (ESRC), REF: AMREF-ESRC P747/2020. We also obtained approvals from National Commission for Science, Technology and Innovation (NACOSTI), REF: NACOSTI/P/20/7726. Approval was also obtained from the Liverpool School of Tropical Medicine (LSTM) and the African Population and Health Research Center (APHRC) internal ethical review committees. All participants provided informed written consent before participating in an interview including consent for using photos and videos if there were any.

## Results

In addressing our two questions, we present two distinct results out of our study. First in addressing the question, what are the benefits of formal and informal actors, we present formal and informal actors with their diverse networks. In addressing the question of how do formal and informal actors complement each other for the benefit of vulnerable groups, we present findings on the complementarity of formal and informal actors.

### Theme 1: Formal and informal support actors and their networks

**Figure 3:**
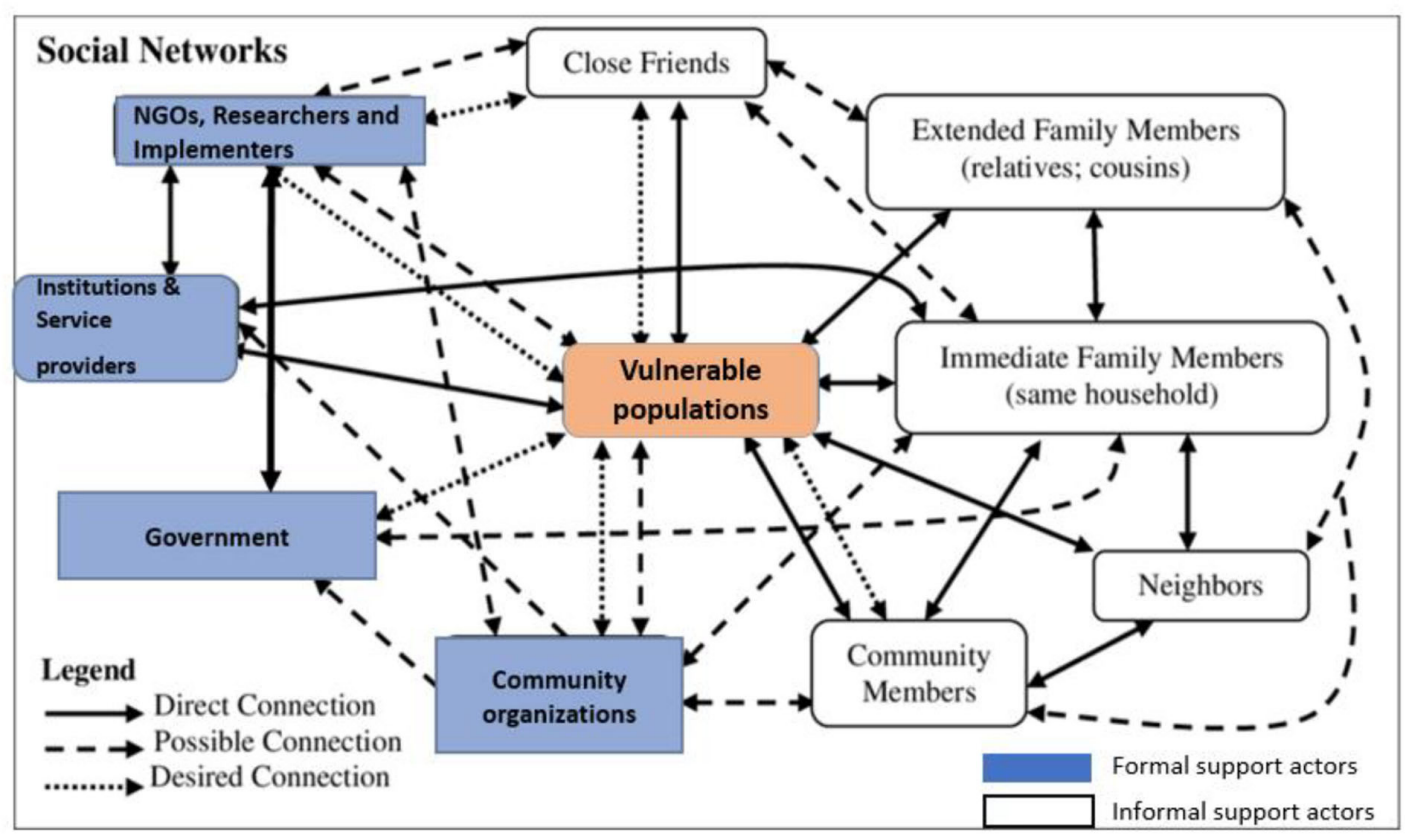
Results of network of actors for vulnerable population.

There was a great deal of shared knowledge about the nature and scope of informal support actors. Participants described how the actors were essential for meeting the health and wellbeing needs of vulnerable populations. Participants described that to support the vulnerable populations, there needed to be functioning support networks, which comprised of direct, possible and desired networks. Informal support actors included family, neighbors, friends and community members and they had much direct role and direct networks. Formal actors included government ministries, community groups, institutions, non-governmental organization and implementers, and they had more of desired and possible networks (Figure 3).

Informal actors who were providing support services for the vulnerable populations directly without using agencies were described to have a direct network, while the formal actors that had the potential of providing support services because they were offering the services in the community were described to have possible networks. There were formal actors who were known by study participants to be of great influence in providing support services but were not felt by the vulnerable groups and were described to have desired networks. Some actors had multiple networks. The network of actors identified by the study participants is summarized in Figure 3.

*“One can possibly have government services but your life cannot be complete unless you have family doing those normal things directly for you like washing clothes, doing the groceries and all those things in the house and everything like that”* (Older person, Male).

We explored further on the formal and informal actors. As such we asked the study participants questions on (a) what are benefits of informal support actors and networks? (b) What are the benefits of formal support actors and networks? This laid the basis for understanding complementarity of formal and informal actors and their networks.

#### Informal support actors and networks have multiple benefits

Study participants described how informal actors and related networks reduced exhaustion and stress, as they support the vulnerable populations in performing some chores that would be strenuous. In addition, participants believed that informal support actors and networks helped change attitudes on vulnerabilities and built personal skills for having to deal with anxiety and depression through experiential learning.

*“When you have a neighbor supporting you, it reduces stress. If there are family members doing some household chores for you, then that gives you relief and reduces being tired”* (PWD, Male).

“*It is the normalizing of old age by neighbors and friends that reduces worries*…*You meet with other older persons who give you tips on how to adjust to life challenges, as they have also experienced at one point…At this age, we cannot survive without friends”* (Older person, Female).

Furthermore, study participants unanimously described how informal networks were defined as well organized and essential. The participants underscored how informal actors and related networks were accessible, available and dedicated to supporting the vulnerable people in the community.

*“The informal actors are very organized, dedicated and they are always available to support. I can say they work for 24 hours”* (Child head of a household, Male*). “I don't know them by names and they have the heart to help. I met that youth when I was depressed, she was going to board a bus but she stopped to help me”* (PWD, Female).

The benefits of informal actors and their networks were seen to be collective and additive, that is, the community grew and developed in its capacity to support vulnerable people. Study participants described how informal actors and networks were helpful in resolving issues that would have been resolved by the formal actors.

*“As years go by and you are not able to do some things, you get to learn about how to seek help from people around…I would say we are going through difficult circumstances but there are volunteer individuals who support us all through. If it were not for neighbors, family and youth groups around to do work that should have been done by government, maybe our situation would be worse”* (Older person, Female).

#### Formal support actors and networks have multiple benefits

Participants did not comment much on the formal actors, as they were not accessible to many vulnerable groups in the informal settlements. However, some of the participants described how formal actors were trained and had expertise on tasks, including but not limited to coordinating support and service provision.

*“The formal actors are trained and know how to do their work more while supporting us”* (Child head of a household, Male).

Few participants who acknowledged the formal actors and networks, described how the actors and networks played a key role in providing informal actors and networks with information and assurance of support. The study participants consistently stated how the actors and networks performed their roles by sharing skills, knowledge, and stories of caring as well as educating informal actors on needs and expectations of vulnerable persons.

*“Formal actors support us directly but in many cases they offer support to our caregivers including training. Sometimes they offer skills and take them through the steps of managing emotions and supporting vulnerable groups like us”* (Older person, Male).

### Theme 2: A complementary relationship between formal and informal actors and their networks

Beyond the multiple benefits of formal and informal actors individually, we find complimentary benefits of their activities. We asked participants to describe how formal and informal actors and networks complement each other. We present the findings below with different complementarities portrayed on roles and responsibilities; rules, regulations and governance; knowledge, skills and family dynamics; information flow to the vulnerable populations; transition of actors; availability and access of actors and involvement.

#### Complementarity on roles and responsibilities

Formal actors and networks appear to currently play a minimal role in mobilizing and supporting vulnerable populations. As such religious institutions, neighbors and some social-specific informal groups and networks played a key role in supporting the vulnerable groups efficiently. Despite the minimal role, formal actors and networks were viewed to be important and powerful for the operations of informal actors.

*“Formal actors and networks play a small role. Quite often it's just friends or church or neighbors who support us on time. The role that formal actors play could be viewed as minimal but they are powerful in the work they do. For example, a policeman here may not be seen frequently, but make the work of informal actors conducive”* (Child-head of a household, Female).

#### Complementarity on rules, regulations, and governance

There were complex rules and regulations that informal actors were operating within. These included privacy and confidentiality. For example, study participants described how informal actors could be aware that vulnerable people needed help, yet due to privacy, they were unable to mobilize the needed support. However, in such cases, formal actors were described to prioritize assistance at the expense of privacy in their attempt to support vulnerable populations.

*“Sometimes a friend, family or neighbor could see an old person in need of help but cannot call out for a better support because they do not like disclosing people's secret. However, the formal actors would forgo privacy and ensure someone get help*” (Older person, Male).

Corruption and misuse of public resources by formal actors was unanimously reported. For example, participants described how relief food and other support for the vulnerable groups, distributed by formal actors mostly benefited non-vulnerable groups. However, where formal actors collaborated with informal actors like the women groups, youth groups or religious leaders, the most vulnerable populations always benefited.

*“They will register You {as individuals to receive aid) but your name will not reflect in the beneficial lists; they will cancel. When the women, youth and religious groups are involved such things related to injustice does not happen…*Some f*ormal leaders want to take all the aid that comes for themselves, and share with their family members because most of their relatives get relief food and us we do not get* (Child-head of a household, Male).

#### Complementarity on knowledge, skills, and family dynamics

Some study participants described how informal support actors and networks were highly problematic due to their lack of skills and family dynamics, as many of the actors were not properly trained in handling vulnerable people. The formal actors on the other hand were skilled and trained on handling vulnerable populations and were described to come in handy when called upon by informal actors. Further, informal structures were described as knowledgeable on community and community activities, whereas the formal structures had expert knowledge. As such, more benefits were realized when both actors and networks worked together in complementarity.

*“There is no training that informal actors go through; they lack knowledge and skills but they offer us counseling here and are handling issues based on convenience and sometimes it can be life-threatening. Formal actors are usually trained and skilled and they are very useful when called upon by informal actors to offer additional support*” (Child-head of a household, Female).

*“Formal actors just do their work. I think they are trained and skilled. However, without informal structures, the formal actors will not understand the community activities”* (PWD, Male).

#### Complementarity on information flow to the vulnerable populations

Participants reported problems of lack of overall coordination and flow of information about what was available for vulnerable persons. As such, informal actors and networks did not always refer vulnerable persons for specialized services in cases where more specialization was needed. Vulnerable groups also lacked information on where to find support from the formal actors and networks and eventually ends up utilizing the informal actors and networks who are known at the community. By itself, some older child heads of households, older persons or PWD would continue suffering for a long period, while negotiating with informal actors and networks to offer space for formal actors and networks, who had clear communication channels and referrals and were dependable.

*“Our family members and neighbors who are helping us also struggle, they have so many questions without answers…what do I do? where do I send this person?, how do I refer?' Where's the nearest social worker? So they end up trying all they could to support us*. (PWD, Female).

*“Formal actors have a communication channel and clear ways of referring someone. They are better than the informal actors and are reliable…Many people here do not know where to go when they need support, as such they resort to neighbor, family and anyone around”* (Child-head of a household, Male).

#### Complementarity on transition of actors

The changing nature of communities and informal actors and networks was a barrier to supporting the vulnerable population. The changes in the community included family income, economic recession, retirement, thus losing social networks and migration. In addition, a belief that if people have strong networks throughout their life will also have strong networks of support in old age and vice versa, brought forth challenges to individuals who were isolated at the time of our study. The formal actors on the other hand were described to have a clear succession plan for takeover on supporting vulnerable populations.

*“There has been a lot of relocation, as such the individuals who already knew our challenges have left. This has affected us…If somebody has lived their life isolated… when it comes to old age that's how they will be isolated”* (PWD, Female).

*“Those support actors who are working under government are better placed because they have a good handover when they retire from work”* (Older person, Male).

#### Complementarity on availability and access of actors

Informal actors and networks were perceived to be easily accessible from the community and were described to be efficient. Whereas, formal actors and networks were not accessible hence not preferred by the vulnerable groups in the informal settlements. As such, informal actors and networks filled gaps in service provision enabling service provision to people who did not have an extensive formal support.

*“Some formal structures come here and initiate some project activities to support us and disappear completely. To be sincere the support activities are not running well. If it were the neighbors or the family members initiating them, they would be here every day following up on how the activities operate”* (Older person, Female).

*“The informal support help us understand a belief that informal actors and networks will always exist to provide support where formal actors and networks do not exist* (PWD, Male).

Study participants described how hierarchies in accessing formal actors for support was highly reported. This is because formally and administratively, some community steps should not be bypassed. The chain in the hierarchies was described to affect efficient access to support services. On the other hand, in cases where efficient services were needed, informal actors and their networks were preferred.

*“We mostly go to the chairman first because you cannot bypass steps. You start from the ground going up to other higher hierarchies in the government. By the time you get the solution, maybe you have suffered much. If you go to neighbors, family member or the religious leaders, you do not have to think of hierarchies while in need of a solution”* (PWD, Male).

*“Sometimes the chain of command is complicated since if you want to go to the chief they will send you to the chairlady, chairman's and so on and you cannot easily access them, and this is the reason why vulnerable people rarely get support”* (Child-head of a household, Female).

#### Complementarity on involvement

Low involvement in the community was also reported to affect the success of formal actors' roles in the community. Support from informal actors on information of who needs support, where and when resulted in formal actors being effective in support for the vulnerable populations.

*“There is this NGO; they came here and stationed sanitation facilities without consulting. We ended up not using them. When youth groups are involved, they usually consult with us and our issues are featured”* (PWD, Male).

This led us to ask for an example of a “best practice” in social networks for formal actors and networks to involve and work with already-established community organizations, or the individuals who are already supporting the vulnerable populations. This is because there were few cases where participants described formal actors working directly with vulnerable populations.

*“All social networks available should be strengthened from all angles by everyone and formal actors and networks should aim at working with groups and individuals who have already started working with us”* (PWD, Male).

In conclusion, empowering the vulnerable groups with information on the support to obtain from formal actors will greatly enhance complementarity of support. Among informal actors, the interactions varied depending on the intimacy and behaviors of the recipients of support. Moreover, it is important to encourage informal actors to make their services complimentary by recording individual and family requirements (i.e. financial or humanitarian or medical support), which could lead to a collaborative process between formal and informal actors.

## Discussion

We explored the complementarity of formal and informal actors and related networks in support of vulnerable groups in informal settlements using governance diaries approach, supported by a multi-pronged ethnographic data collection processes which included participant diaries, IDIs, informal discussions, observations and reflective sessions. The key findings highlight the need to critically question the assumptions and rationale that lie behind the existence of dichotomous formal and informal support actors in silos as it relates to vulnerable populations in informal settlements. This is because complementarity of actors in delivery of services best captures the current relationship between the formal and informal support actors (3). Study participants described how informal actors and networks provided support to vulnerable persons because the formal actors and networks were non-existent or near-absent in service delivery in informal settlements. Our results showed that informal actors and networks are essential as they provide both task-oriented and emotional support to vulnerable persons. The informal actors and networks were considered to be naturally forming, organized, and unique in supporting the needs of the vulnerable populations in many cases (6). Our findings described how formal actors provided professional and specialized support as they were trained and skilled and at times supported the informal actors to offer better support.

Our results also showed how informal actors, though filling important service delivery gaps, were identified as highly problematic as they were not qualified and had difficulties working. These challenges underscore their inability to replace formal actors and call for at least the complementing roles of formal actors and related networks. This dimension is related to other studies describing the need to exercise caution to avoid assumptions that formal support does not matter since providing formal social support is essential in support of the vulnerable population (2, 6). While this is what formal actors are statutorily tasked to do, this is an important finding in our study context, as formal actors are usually seen as oblivious or insensitive to the needs of the vulnerable populations in informal settlements (7, 53). Notwithstanding, evidence on complementarity suggests that the public approach to the social support of the vulnerable population through informal structures is uncommon or yet to be embraced and utilized by the majority of formal structures (5, 6). The implication is that while there may be tensions between formal and informal actors and networks, our results suggest that participants would welcome guidance and resources to enable them to operationalize social support for vulnerable populations through complementarity of functions of formal and informal actors and networks. This is supported by other studies, describing that social support is perceived to be helpful and individuals need guidance on operationalization (2, 5).

Participants were already positively disposed to the benefits of both formal and informal actors and networks and this included reaping both community knowledge and expert knowledge. This is a profound finding and presents itself as an opportunity for utilizing dual knowledge in supporting the vulnerable persons in the community. It counters the narrative which places services from formal actors as outside of the community, as somehow different and less trusted (54, 55). In reality, some formal actors and networks are sometimes members of the community (2, 5, 6), lived, worked and available to play roles as informal actors and networks in support of vulnerable populations. Another implication is the clear scope we found for formal actors and networks to target and support informal actors and networks through already existing community groups such as schools, religious institutions, and social clubs thus developing partnerships, community capacity and consequent increased benefits to the vulnerable groups (37). This is because the formal actors and networks are usually called upon when the informal support have exhausted their capability and the situation of the vulnerable has deteriorated so much that the informal network requires assistance to cope or critical elements of the informal network are missing. Our findings are speaking to a wider emerging evidence of significant improvements in health outcomes among slum dwellers in Nairobi within relatively short periods, following interventions that promoted collaboration and cooperation of multiple actors, including formal and informal service delivery structures. While not primarily focusing on complementarity, an intervention research program on maternal and child health—the Partnership for Maternal, Newborn and Child Health (PAMANECH) in Korogocho and Viwandani slums—implemented between 2012 and 2016 increased the capacity of private health facilities in the slums to provide basic emergency obstetric care and significantly reduced home deliveries, by bringing together local health service providers and officials of Ministry of Health in a complimentary than adversarial relationship (56). Further, as part of the search for pathways to address the challenge of health inequity between slum and non-slum urban dwellers, another study highlights the complementary roles of mobile outreach services and implementation research studies in expanding healthcare access to hard-to-reach but vulnerable groups in informal settlements beyond public facilities and other traditional service delivery models (57, 58). It suggests like our current study, the potential promise of nontraditional service delivery models that are convenient and adaptable to specific contexts in bridging service access and utilization deficits among disadvantaged populations.

## Conclusion

In line with the present findings, we conclude that informal actors and networks for social support of vulnerable populations are needed regardless of the availability of formal actors and networks more so in the informal settlements. This is because there is a prevailing near absence of many critical formal support in informal settlements and so informal actors and networks have devised support network mechanisms. Our findings underscore the need for a consequential engagement with informal stakeholders and incorporation of their rich local intelligence, often ignored and generally not quantified, documented, harnessed, nor incorporated into policy and action. Nevertheless, mechanisms that entails a complementarity of formal and informal actors and related networks are essential to support vulnerable persons because informal structures are knowledgeable on community and community activities, whereas the formal structures have expert knowledge. Formal actors and related networks should establish, support, or maintain the informal actors and related networks through goodwill and broader scope. Shifting the focus of complementarity of formal and informal actors and networks from a point of control to autonomy and collaboration would be a significant step in empowering vulnerable groups to make decisions about where to seek support. Comprehensive education and skills development that enables vulnerable groups and their caregivers to determine where, how, when and why to seek support from both formal and informal support actors and networks, and that seeks to ensure informed decision-making—is the best antidote to advancing support to the vulnerable populations in informal settlements. Lack of support can only persist in the context of misinformation and darkness, which is why efforts to raise awareness are essential and need to go further.

On the global agenda, our findings of complementarity speaks to calls for not only breaking down traditional silos but building more cross-sectoral decision-making and multi-stakeholder partnership approaches and links among sectors, an era ushered by the SDGs 2030. It is typified by joint actions by health and other government sectors, representatives from private, voluntary and non-profit groups, to improve the health of populations, with actions taking the forms of cooperative initiatives, alliances, coalitions or partnerships and co-creation of solutions. These lend impetus for the need to extend the tradition in seeking complementarity between formal and informal structures in addressing the challenges of vulnerability and marginality among the urban poor; a call consistent with incorporating rich local intelligence, adaptive capacity and survival instincts as integral part of the building blocks for policy and action in the region and beyond.

## Data availability statement

The data presented in this study is available on request from the corresponding author. Other data related to this study is not publicly available because it is included within upcoming articles and cannot be shared at this time.

## Ethics statement

This study was conducted in accordance with the Declaration of Helsinki, and approved by the AMREF Health Africa's Ethics Scientific Review Committee (ESRC), REF: AMREF-ESRC P747/2020. A research permit from National Commission for Science, Technology and Innovation (NACOSTI), REF: NACOSTI/P/20/7726 was also obtained. Approval was also obtained from the Liverpool School of Tropical Medicine (LSTM) and the African Population and Health Research Centre (APHRC) internal ethical review committee. All participants provided informed written consent before participating in an interview including consent for using photos and videos if there were any. Written informed consent from the participants' legal guardian/next of kin was not required to participate in this study in accordance with the national legislation and the institutional requirements.

## Author contributions

Conceptualization: IC, CK, and BM. Data curation, formal analysis, and writing–original draft: IC. Methodology and writing–review and editing: IC, CK, AS, EI, and BM. All authors approved the manuscript for submission.
